# Supplementary material for: Evolution﻿ of spin freezing transition and structural, magnetic phase diagram of Dy2-xLaxZr2O7 (0 ≤ x ≤ 2.0)
Source: Sci Rep. 2021 Oct 6;11:19832. doi: 10.1038/s41598-021-99035-z (PMC8494935; doi:10.1038/s41598-021-99035-z)
Supplement: Supplementary file 1 — Supplementary Information. [file 41598_2021_99035_MOESM1_ESM.pdf]

Evolution of spin freezing transition and structural, magnetic phase diagram of $Dy_{2-x}La_xZr_2O_7$ ($0 \leq x \leq 2.0$)

Sheetal* and C. S. Yadav
School of Basic Sciences, Indian Institute of Technology Mandi, Mandi-175005 (H.P.), India

A. Structural studies

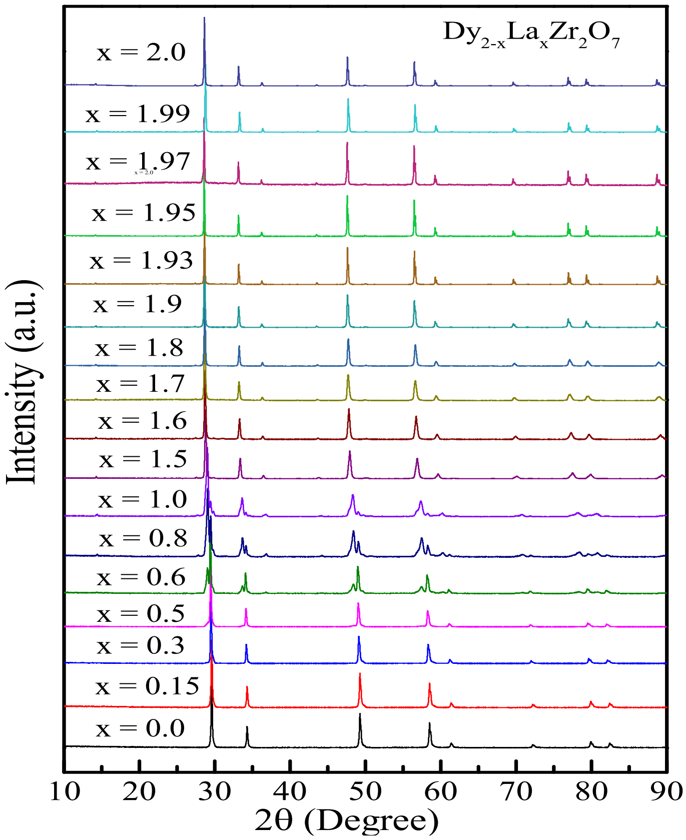

Figure S1: Room temperature x-ray diffraction pattern of $Dy_{2-x}La_xZr_2O_7$ (x = 0.0 - 2.0). A clear shift in peak positions towards lower θ values indicates the increase in lattice constant with increase in the La concentration.

Figure S1 and S2 show the x-ray diffraction pattern of $Dy_{2-x}La_xZr_2O_7$ (x = 0 - 2). The XRD patterns of the compounds are fitted nicely in Fd$\bar{3}$ space group and show the single phase of these compounds. Structural evolution from weak pyrochlore phase ($0 \leq x \leq 0.5$) to completely stabilised pyrochlore phase ($1.5 \leq x \leq 2$) is clear from the x-ray studies. The observation of the superstructure peaks for $x \geq 0.5$ directs the structural geometry towards the stable pyrochlore phase. The refined parameters for all the compositions are listed in Table I. Fig. 3 shows the refined x-ray pattern of a mixed-phase compound $DyLaZr_2O_7$, where XRD data is nicely fitted with the parameters of disordered fluorite and pyrochlore phase using the same space group and three different lattice parameters. Though the majority phase ($\sim$ 97%) is pyrochlore (a = 10.6669 Å), there are two other $\sim$ 2% and $\sim$ 1% weak pyrochlore phases with lattice parameters a = 10.4496(3) Åand a = 10.3737(3) Å.

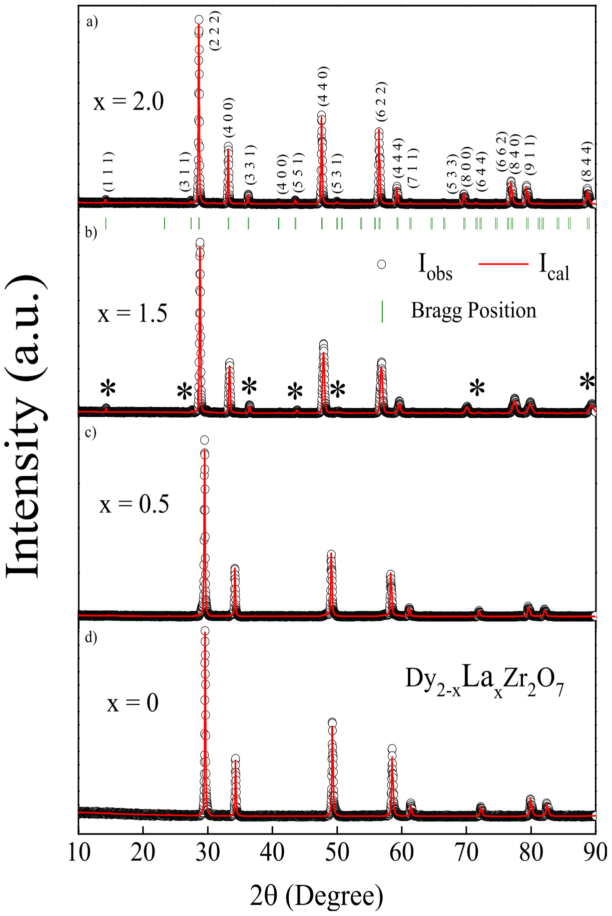

Figure S2: The Rietveld refined x-ray diffraction pattern of $Dy_{2-x}La_xZr_2O_7$ (x = 0, 0.5, 1.5, 2.0) using Fd$\bar{3}m$ space group. Peaks marked by star are the superstructure peaks belonging to pyrochlore structure, and indicates the pyrochlore type ordering in the compounds.

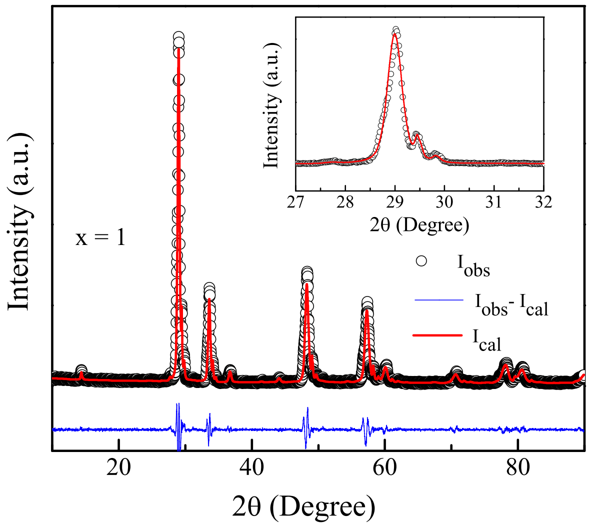

Figure S3: The Rietveld refined x-ray diffraction pattern of $DyLaZr_2O_7$. Inset shows the splitting of main peak and refinement with disordered fluorite and pyrochlore phase more clearly.

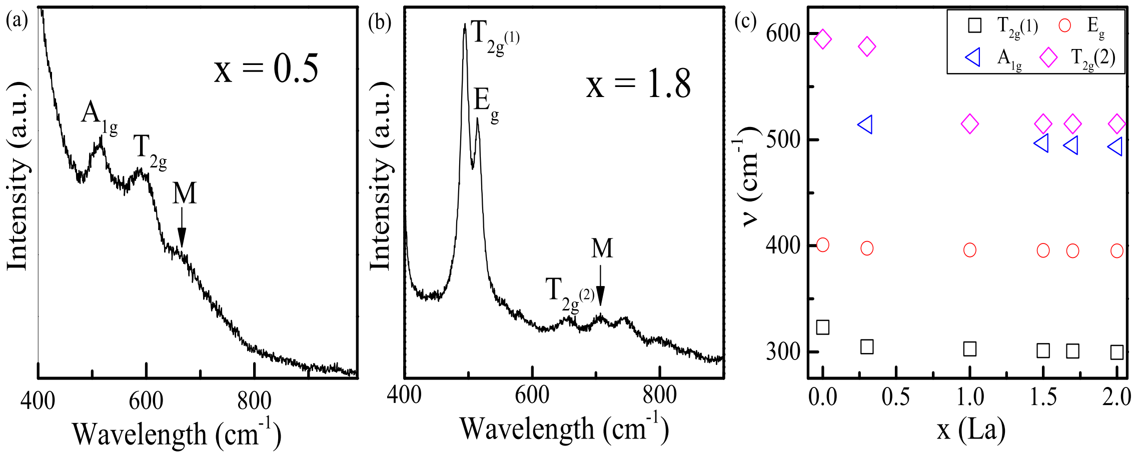

Figure S4: (a & b) Room temperature Raman spectra of $Dy_{2-x}La_xZr_2O_7$; (0.5, 1.8) showing the presence of Raman modes corresponding to pyrochlore structure. (c) Variation in the phonon frequency of Raman modes with increase in La concentration.

B. Magnetic studies

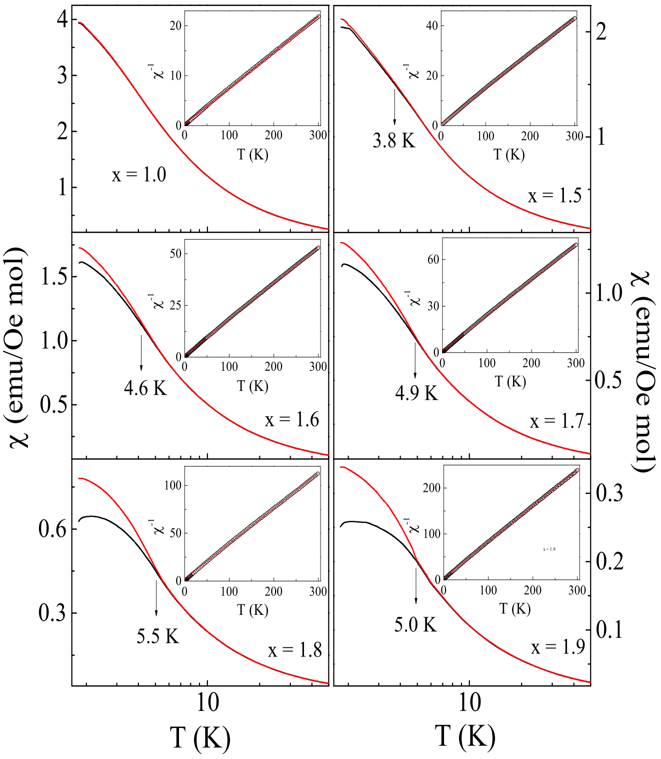

Figure S5a: Dc magnetic susceptibility χ_{dc} (ZFC and FC) versus T at H = 100 Oe for $Dy_{2-x}La_xZr_2O_7$ ($0 \leq x \leq 2$). Inset: Curie-Weiss fit of the magnetization data at 100 Oe in the temperature range 30 - 300 K.

Figures S5a and S5b shows the dc magnetization data of $Dy_{2-x}La_xZr_2O_7$ ($1.0 \leq x \leq 1.99$) and the inset of each plot shows the CW fit of the inverse susceptibility data. The inverse susceptibility data of $Dy_{2-x}La_xZr_2O_7$ (measured at H = 100 Oe) is fitted with Curie-Weiss law in the high temperature regime ($T \geq 30$ K) using the relation $\chi = C/(T - \theta_{cw})$, where C and θ_{cw} stands for Curie constant and Curie-Weiss temperature respectively. From the Curie constant, the effective magnetic moment was calculated using $\mu_{eff} = \sqrt{3k_B C/N_A}$, where N_A is the Avogadro's number. The obtained value of θ_{cw} and μ_{eff} for all the substituted compounds are listed in Supplementary Table II. The negative value of θ_{cw} signifies the dominance of AFM interactions in the system. Fig. S6 shows the variation in the bifurcation point (T_{irr}) with La substitution and the magnetic field. Fig. S7 shows the temperature dependence of real and

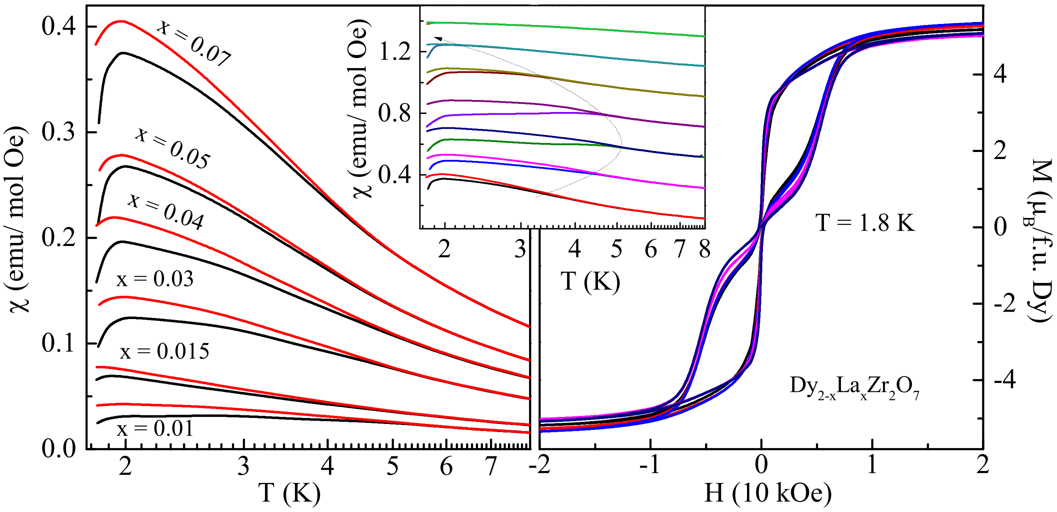

Figure S5b: (Left) Dc magnetic susceptibility χ_{dc} (ZFC and FC) versus T at H = 100 Oe and (Right) isothermal magnetization for $Dy_{2-x}La_xZr_2O_7$ ($1.93 \leq x \leq 1.99$). Inset: ZFC and FC data of x = 1.93 to show the field dependence of T_{irr}.

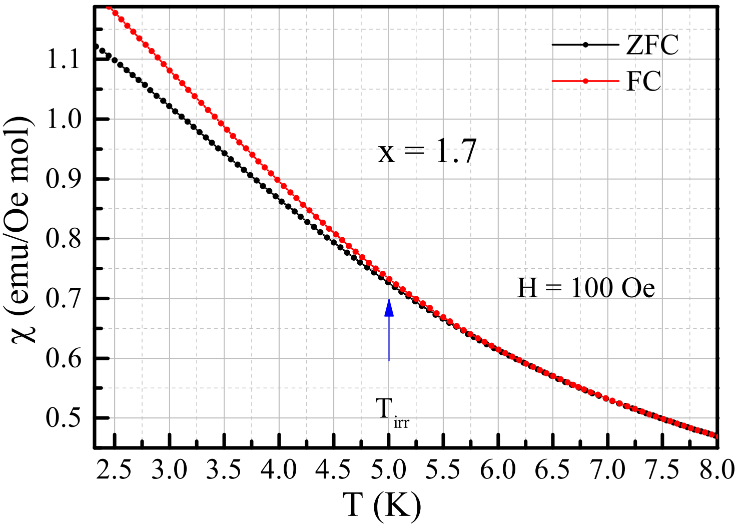

Figure S5c: Temperature dependence of magnetization data for x = 1.7 composition. The point of visible separation between zfc and fc curve is identified as T_{irr}. However there can be an error of ± 0.2 K in T_{irr} for these compounds.

imaginary parts of ac susceptibility measured at various frequencies between 10 - 1000 Hz. A clear relaxation peak starts evolving on entering the biphasic region and becomes prominent in the stable pyrochlore phase. In contrast, parent compound $Dy_2Zr_2O_7$ exhibits a different behavior and paramagnetic down to 1.8 K. The magnetic phase transition is consistent with the structural phase transition (discussed in the main text). The ac susceptibility data is analyzed by Cole-Cole plot (Fig. S8 (left)) and Arrhenius fit (Fig. S8 (right)), and the obtained parameters are plotted in the main text. To further analyze the relaxation behavior in detail, ac susceptibility measurements were performed as a function of frequencies for T < 40 K and are shown in Fig. S9.

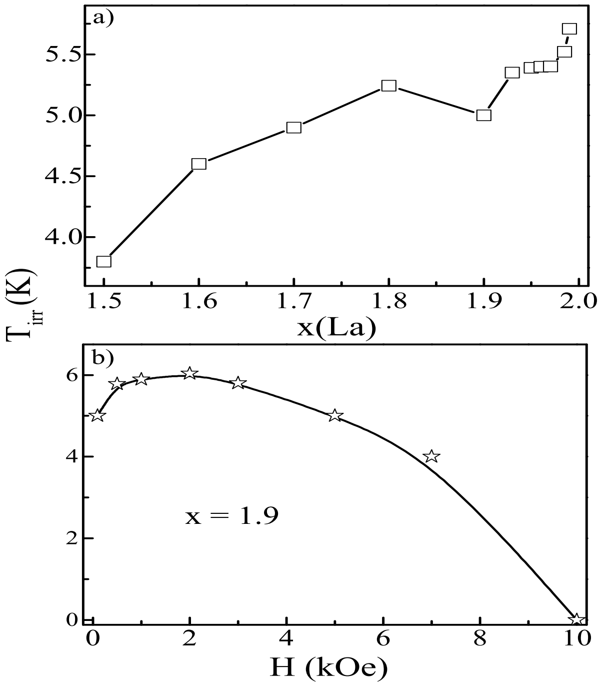

Figure S6: (a) La concentration dependence of T_{irr} taken as a bifurcation point of ZFC and FC data in $Dy_{2-x}La_xZr_2O_7$ ($1.5 \le x \le 1.99$). (b) Field dependence of T_{irr} measured for $Dy_{0.1}La_{1.9}Zr_2O_7$.

TABLE I: Magnetic parameters of $Dy_{2-x}La_xZr_2O_7$; $0.0 \le x < 2.0$

x(La)	μ_{eff} (μ_B)	θ_{cw} (K)	p	Ref
0	16.4 (1.09)	-9.6(2)	-	[1]
0.3	14.12 (0.65)	-7.14(1)	-	,,
0.5	12.94 (0.43)	-7.02(3)	-	This study
1.0	10.51 (0.35)	-5.63(2)	-	,,
1.5	7.56 (0.26)	-6.61(2)	0.80	,,
1.6	6.80 (0.23)	-5.64(2)	0.71	,,
1.7	5.93 (0.19)	-5.80(2)	0.66	,,
1.8	4.67 (0.18)	-6.63(7)	0.64	,,
1.9	3.21 (0.09)	-6.17(5)	0.6	,,
1.93	2.83 (0.05)	-6.11(3)	0.25	,,
1.95	2.39 (0.03)	-6.05(1)	0.26	,,
1.96	2.22 (0.025)	-6.21(3)	0.27	,,
1.97	1.80 (0.017)	-4.41(3)	0.26	,,
1.985	1.25 (0.013)	2.89(2)	0.28	,,
1.99	0.84 (0.009)	2.81(6)	0.26	,,

[1] S. Devi, A. Ali, S. Rajput, Y. Singh, T. Maitra, and C. Yadav, Journal of Physics: Condensed Matter **32**, 365804 (2020).
[2] J. G. A. Ramon, C. Wang, L. Ishida, P. Bernardo, M. Leite, F. M. Vichi, J. Gardner, and R. Freitas, Physical Review B **99**, 214442 (2019).

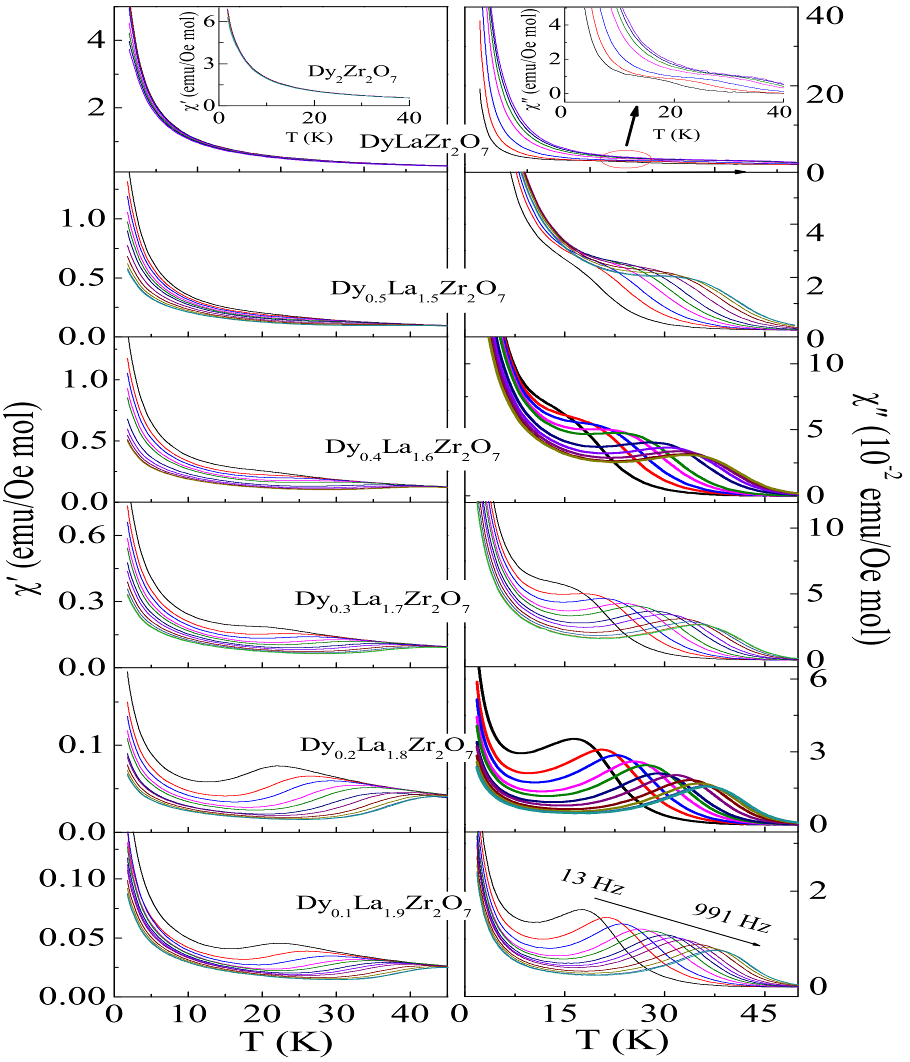

Figure S7: Temperature dependence of real and imaginary part of ac susceptibility for $Dy_{2-x}La_xZr_2O_7$ ($0 \leq x \leq 1.9$) at various frequencies ranges between 10 - 1000 Hz. Left inset shows the temperature response of χ' of $Dy_2Zr_2O_7$ and right inset shows the temperature response of χ'' for $DyLaZr_2O_7$.

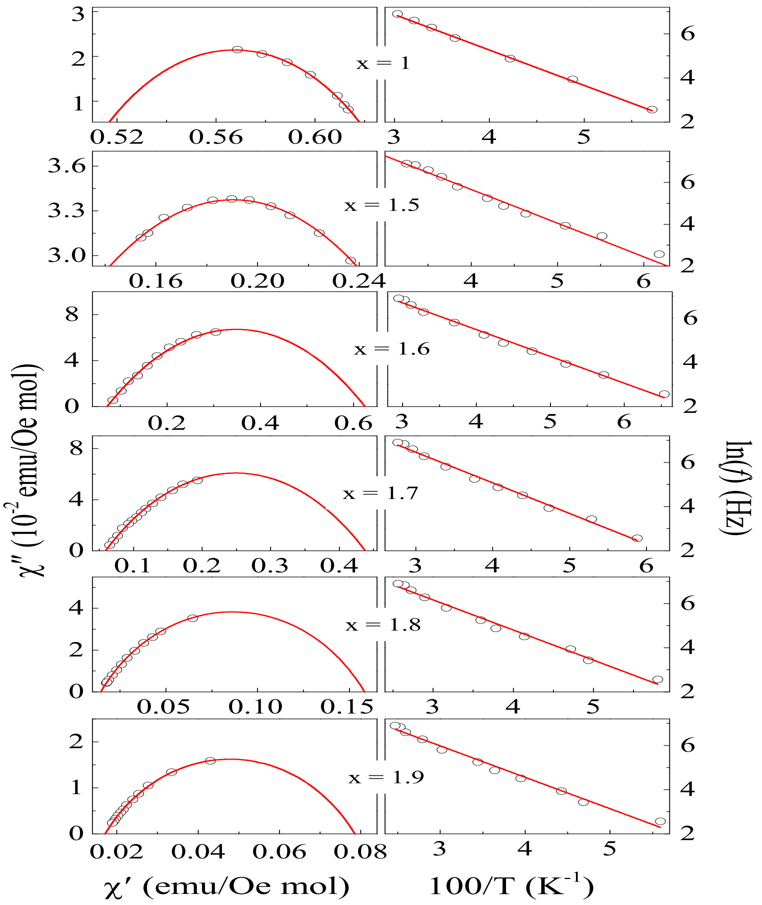

Figure S8: Cole-Cole plot of χ' and χ'' data around the freezing temperature and Arrhenius fit of $Dy_{2-x}La_xZr_2O_7$ ($1.5 \leq x \leq 1.99$).

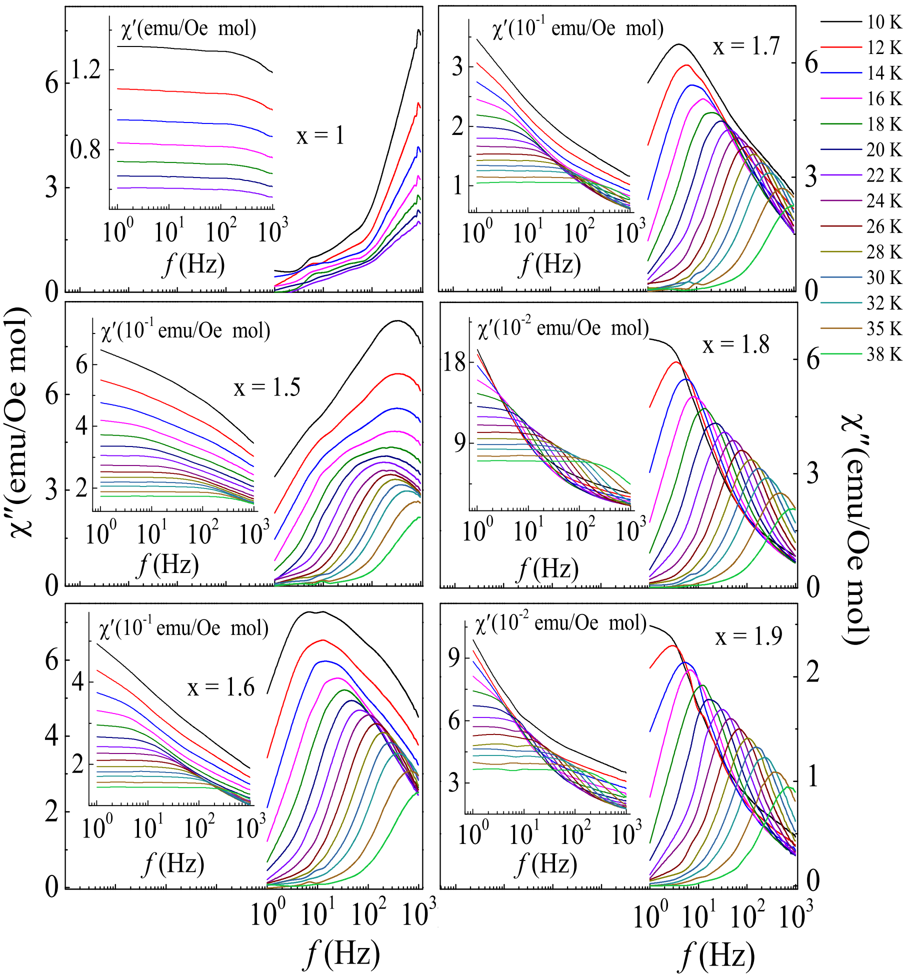

Figure S9: Frequency dependence of the imaginary part of ac susceptibility of $Dy_{2-x}La_xZr_2O_7$ ($1.0 \leq x \leq 1.9$) below and above the freezing temperature in zero applied field. The presence of prominent single peak is the signature of single characteristic relaxation time.

TABLE II: Structural parameters $Dy_{2-x}La_xZr_2O_7$; $0.0 \leq x \leq 2.0$

x(La)	r_A/r_B	χ^2	x(O)	a(Å)	Phase%	Ref
0.0	1.43	1.46	0.3667(23)	10.4511(3)	100	[1]
0.15	1.44	2.26	0.3682(28)	10.4832(4)	100	,,
0.3	1.45	2.22	0.3694(29)	10.4972(3)	100	,,
0.5	1.47	1.51	0.3710(2)	10.5521(6)	100	This study
0.6	1.48	2.15	0.3271(13)	10.5994(4)	60	,,
			0.3729(3)	10.4797(3)	35	,,
			0.3492(2)	10.3895(6)	5	,,
0.8	1.49	1.95	0.3273(10)	10.6482(5)	88	,,
			0.3604(3)	10.4992(2)	8	,,
			0.3724(3)	10.3889(8)	4	,,
1.0	1.52	2.85	0.3248(7)	10.6669(3)	97	,,
			0.3630(4)	10.4992(4)	2	,,
			0.3730(4)	10.3735(2)	1	,,
1.5	1.56	1.70	0.3291(4)	10.7445(3)	100	,,
1.6	1.57	1.36	0.3205(5)	10.7633(3)	100	,,
1.7	1.58	1.43	0.3287(5)	10.7786(3)	100	,,
1.8	1.59	1.23	0.3281(4)	10.7812(1)	100	,,
1.9	1.60	1.49	0.3285(5)	10.7903(1)	100	,,
1.93	1.604	1.57	0.3287(4)	10.7958(3)	100	,,
1.95	1.606	1.63	0.3282(7)	10.7976(1)	100	,,
1.96	1.607	1.55	0.3287(8)	10.7981(2)	100	,,
1.97	1.608	1.61	0.3295(6)	10.7983(1)	100	,,
1.985	1.609	1.52	0.3282(7)	10.7989(2)	100	,,
1.99	1.610	1.46	0.3282(4)	10.8005(2)	100	,,
2.0	1.611	1.40	0.3277(5)	10.8008(1)	100	,,
